# Creating Semiconducting Polymer Dots with Enhanced Performance Through a Simple Mixed Antisolvent Approach

**DOI:** 10.3390/bios16060308

**Published:** 2026-05-27

**Authors:** Dingshi Xu, Xuehan He, Yi Zhao, Jiasi Wang, Lei Chen

**Affiliations:** 1 Guangdong Provincial Key Laboratory of Sensor Technology and Biomedical Instrument, School of Biomedical Engineering, Shenzhen Campus, Sun Yat-sen University, Shenzhen 518107, Chinawangjs8@mail.sysu.edu.cn (J.W.); 2 School of Pharmaceutical Sciences, Shenzhen Campus, Sun Yat-sen University, Shenzhen 518107, China

**Keywords:** semiconducting polymer dots, nanoprecipitation, antisolvent, core–shell, Förster energy transfer

## Abstract

We present an optimized method for producing semiconducting polymer dots using a water–ethanol mixed antisolvent during nanoprecipitation. Compared to conventional Pdots made with pure water as the antisolvent, these newly produced Pdots exhibit simultaneously enhanced fluorescence efficiency and stability of particle size and emission spectra. These findings should be mainly attributed to an improved core–shell Pdots nanostructure formed by a sequential nanoprecipitation process. It offers Pdots a purer, more compact, and hydrophobic inner core, coated with a greater number of hydrophilic polyethylene glycol shells. This viewpoint is further reinforced by Förster energy-transfer efficiency in a fluorescence donor-acceptor Pdots system. The novelly prepared Pdots can better encapsulate small-molecular cargoes and more efficiently bioconjugate to targets. Consequently, it demonstrates improved specific immunofluorescence staining of microtubule structures in living cells.

## 1. Introduction

Over the past decade, semiconducting polymer dots (Pdots) have attracted significant academic interest for biological applications, including in vitro cell imaging and biosensing, as well as in vivo tissue imaging and treatment [1,2,3,4,5,6,7,8,9,10,11,12,13]. Semiconducting Pdots are defined as a type of conjugated polymer (CP)-based nanoparticles (NPs) with small particle sizes (≤30 nm) and high CP content (≥50%) [14]. Most reported Pdots are produced through the classic nanoprecipitation approach [15,16]. Generally, to obtain a Pdots aqueous solution, a tetrahydrofuran (THF) solution containing CPs and a non-conjugated amphiphilic polymeric surfactant is injected into pure water antisolvent. Then the organic THF solvent is purged with N_2_ gas under heating. The non-conjugated amphiphilic polymers are typically functionalized polyethylene glycol (PEG)-based block or graft copolymers, containing both hydrophobic and hydrophilic segments, such as the commonly used polystyrene-polyethylene glycol-carboxylic acid (PS-PEG-COOH) and distearoyl-N-(3-carboxypropionoyl poly(ethylene glycol)succinyl)phosphatidylethanolamine (DSPE-PEG-COOH) polymers [17,18]. They are added to prevent the bare CP-based NPs from self-aggregating, which enhances the colloidal stability of Pdots in aqueous solutions. As a result, the obtained Pdots solution can stay stable and clear without coagulation for several months [14]. Additionally, the nonspecific adsorption of Pdots on biological tissues or cells can be effectively decreased by the polymeric surfactant [19]. Pdots obtained in this technique are particularly suited for bio-applications due to their low cytotoxicity and appropriate nano-size [20,21,22,23].

When the excellent solvent (THF) evaporates during the nanoprecipitation process, the solubility of the polymers plummets quickly, and both CPs and amphiphilic polymers will immediately come together through hydrophilic-hydrophobic interaction to create Pdot NPs. Theoretically, Pdots have a core–shell nanostructure, where the core is made up of hydrophobic segments (photoactive CPs and the hydrophobic portion of the amphiphilic surfactant) that congregate at the micelle’s center and face away from the water dispersion medium, and the shell is made up of hydrophilic segments of the amphiphilic polymer that protrude into the water dispersion medium. These core–shell nanostructured Pdots can encapsulate medicines, dyes, catalysts, and other visiting molecules [24,25,26,27,28]. Furthermore, biomolecules can be used to modify their surfaces [29]. Both are crucial for the imaging, sensing, and therapy of biological systems.

Do Pdots really possess an ideal core–shell nanostructure as described above? Most likely, the answer is no. The actual composition distribution of Pdots is still uncertain. The physically encapsulated dyes can easily leak out from Pdots, leading to unstable fluorescence spectra. To eliminate or ease this issue, the dye should be chemically grafted to the side chain of PS-PEG-based surfactant polymer or using poly(styrene-co-maleic anhydride) (PSMA) as an amphiphilic surfactant to generate cross-linked Pdots [30,31,32]. In addition, based on our observations, traditional Pdots are susceptible to swelling at high temperatures.

According to these findings, the Pdots prepared by the traditional nanoprecipitation method may not have a perfect core–shell structure with a compact hydrophobic core. The core may have partially embedded hydrophilic chains, making it somewhat loose in aqueous solution. When employing Pdots as a nanocarrier for dye or medicine cargoes, the leakage problem of physically blended small molecules is undesirable. Additionally, suppose a portion of the hydrophilic PEG chains and functionalized end groups is encapsulated in the hydrophobic core. It is expected to reduce the efficacy of surface chemical modification with target molecules. Exploring the use of Pdot probes for real-time quantitative fluorescence polymerase chain reaction (PCR) applications is somewhat hindered by their unstable nano-size and emission spectra at disparate temperatures. As a result, it is advantageous to optimize the preparation procedure in addition to the material design to produce Pdots with the desired nanostructure and improved optical characteristics [33,34]. And we also need to learn more about the relationship between macroscopic properties and the microstructure of the Pdot NPs.

In this research, we improved the Pdots production method by substituting pure water with a water–ethanol mixed antisolvent. This modification can be easily incorporated into current Pdots manufacturing processes. The Pdots produced using this new method demonstrate enhanced fluorescence efficiency and thermal stability of the nano-sized and fluorescent spectra. These superior properties are likely due to an improved core–shell nanostructure featuring a denser inner core. To validate this hypothesis, we further developed a ternary PFBT-R polymer by embedding a green chromophore within a PF-based conjugated polymer backbone and attaching a red emitter to the side chain. We then examined the fluorescence resonance energy transfer (FRET) efficiency between the green donor (D) and red acceptor (A) in PFBT-R Pdots prepared by both methods. The results showed that the newly produced PFBT-R Pdots exhibit more efficient FRET between the D-A pairs, indirectly confirming a more compact inner core. Additionally, dye-leakage experiments demonstrated that the new Pdots have superior encapsulation ability for small molecular cargoes. They also show enhanced bioconjugation efficiency with the streptavidin (SA) target molecule, resulting in improved immunofluorescence labeling of the cytoskeleton using a Pdots-SA-Biotin-antibody–antigen system.

## 2. Experimental Section

### 2.1. Materials

The conjugated polymers poly(9,9-dioctyl-2,7-fluorene) (PFO), poly(9,9-di-(2′-ethylhexyl)-2,7-fluorene) (PFEH), and poly(9,9-dioctylfluorene-alt-benzothiadiazole) (PFBT) were obtained from American Dye Source Inc. (Quebec, QC, Canada). The amphiphilic polymer, polystyrene grafting with carboxyl-group-functionalized ethylene oxide (PS-PEG-COOH), was purchased from Polymer Source Inc. (Quebec, QC, Canada). DSPE-ICG was obtained from Xi’an Ruixi Biological Technology Inc. (Xi’an, China). 9,9-Dioctylfluorene-2,7-bis (trimethylene boronate), 9,9-Dioctyl-2,7-dibromofluorene, and 4,7-dibromobenzothiadiazole were purchased from Sigma-Aldrich (St. Louis, MO, USA) and used without further purification. The red dye-containing monomer (Monomer-R) has been reported in our previous work [35].

### 2.2. Synthesis of PFBT-R Polymer

A mixture of 9,9-dioctylfluorene-2,7-bis(trimethylene boronate) (0.2792 g, 0.500 mmol), 9,9-Dioctyl-2,7-dibromofluorene (0.2430 g, 0.445 mmol), 4.7-dibromobenzothiadiazole (0.0146 g, 0.05 mmol), Monomer-R (0.0066 g, 0.005 mmol), and Pd(PPh_3_)_4_ (10 mg) under argon was added degassed 2 M aqueous K_2_CO_3_ (2 mL) and degassed toluene (6 mL). The resulting mixture was stirred in the dark at 90 °C for 48 h, and then sequentially end-capped with 0.1 M phenylboronic acid (2 mL) and bromobenzene (1 mL), stirring for 12 h for each addition. After cooling, the reaction mixture was poured into methanol and filtered. The precipitate was collected, dissolved in CH_2_Cl_2_, washed with water, and dried with anhydrous Na_2_SO_4_. After evaporating most of the solvent, the residue was precipitated from stirred methanol to obtain a red fibrous solid with a yield of 55%. The number-average molecular weight (Mn) of PFBT-R is 1.1 × 10^4^ Da, as measured by gel permeation chromatography (GPC).

### 2.3. Preparation of Pdots

#### 2.3.1. Preparation of Pdots Using Pure Di-Water Antisolvent

The traditional w-series PFO, PFBT, PFEH, or PFBT-R Pdots were individually prepared using the corresponding fluorescent semiconducting polymer. Initially, stock solutions of PFO, PFBT, PFEH, PFBT-R, and the amphiphilic polymer PS-PEG-COOH were prepared separately by dissolving each polymer in THF at a concentration of 1.0 g L^−1^. Subsequently, the stock solutions were diluted to prepare a mixture comprising a specified fluorescent polymer—either PFO, PFBT, PFEH, or PFBT-R—at a concentration of 0.08 g L^−1^, along with the PS-PEG-COOH copolymer at 0.02 g L^−1^. This dilution yielded a total polymer concentration of 0.1 g L^−1^ in a 10 mL THF solution. A volume aliquot between 1 and 5 mL of the solution was immediately injected using a pipette in a single-step addition, rather than dropwise, into 10 g of deionized water antisolvent, under sonication using a Branson 2800 ultrasonic device operating at 110 W and 40 kHz in sonic mode. Following continuous 1 min of ultrasound treatment, place the 20 mL sample bottle containing the mixture onto the heating stirrer plate. The THF solvent was then removed by purging the mixture with nitrogen gas at 70 °C for approximately 30 min until the solvent volume was reduced to approximately 8.0 g. The resulting Pdots suspension was sonicated for an additional 1 to 2 min and then filtered through a 0.22 μm cellulose membrane to remove aggregates, producing the final Pdots with concentrations ranging from 10 to 50 μg mL^−1^. A concentration of 60 μg mL^−1^ Pdots was achieved by further concentrating the 50 μg mL^−1^ suspension through solvent evaporation. Typically, a 10 μg mL^−1^ Pdots solution was used for common characterizations such as photophysical properties, DLS, and TEM, while a 20 μg mL^−1^ concentration was employed for cell labeling and imaging. For cell viability assays, Pdots concentrations between 10 and 60 μg mL^−1^ were utilized.

#### 2.3.2. Preparation of Pdots Using Mixed Antisolvent

The new w/e-series PFO, PFBT, PFEH, or PFBT-R Pdots were individually prepared utilizing the corresponding fluorescent semiconducting polymer following the previously described injection and sonication protocol. The difference is that the antisolvent is 10 g mixed antisolvent (5 g of Milli-Q water (Millipore, Darmstadt, Germany) + 5 g of ethanol). Subsequently, organic THF and ethanol solvents were then removed by blowing nitrogen gas into the solution at 70 °C for around 30 min until the remaining solution was ~5 g. An additional 5 g of deionized water was then introduced, and the evaporation process was repeated to further eliminate ethanol. The resulting Pdots solution was diluted to a final volume of 8 mL, subjected to sonication for 1–2 min, and filtered through a 0.22 μm cellulose membrane filter to remove aggregates, yielding the final Pdots with concentrations ranging from 10 to 50 μg mL^−1^. A concentration of 60 μg mL^−1^ Pdots was achieved by further concentrating the 50 μg mL^−1^ suspension through solvent evaporation. Typically, a 10 μg mL^−1^ Pdots solution was used for common characterizations such as photophysical properties, DLS, and TEM, while a 20 μg mL^−1^ concentration was employed for cell labeling and imaging. For cell viability assays, Pdots concentrations between 10 and 60 μg mL^−1^ were utilized.

#### 2.3.3. Preparation of DSPE-ICG Doped Pdots

DSPE-ICG doped w-series Pdots were obtained via the aforementioned reprecipitation method. The fluorescent semiconducting polymer (PFH) and the amphiphilic polymer PS-PEG-COOH were separately dissolved in tetrahydrofuran (THF) to prepare stock solutions at a concentration of 1.0 g L^−1^. Subsequently, the stock polymer solutions were diluted with the corresponding weight ratios of 0.06 g L^−1^ fluorescent polymer, 0.02 g L^−1^ DSPE-ICG, and 0.02 g L^−1^ PS-PEG-COOH copolymer to prepare a 10 mL THF solution mixture with a total polymer concentration of 0.1 g L^−1^. Under sonication, 2 mL of the solution mixture was rapidly injected into 10 g of pure water antisolvent. The solution was then purged with nitrogen gas at 70 °C for approximately 30 min until the remaining solution was reduced to about 8.0 g, effectively removing the organic THF solvent. The resulting Pdots solution was sonicated for 1–2 min and filtered through a 0.2 μm cellulose membrane filter to obtain the final Pdots solution. The DSPE-ICG doped w/e-series Pdots were prepared using a similar process using water/ethanol mixed antisolvent as described in Section 2.3.2.

### 2.4. Apparatus for Pdot Characterization

The molecular weight of the polymer was determined by GPC using a SHIMADZU LC-20AD liquid chromatography instrument with polystyrene as a standard. The particle size of Pdots in aqueous solution was characterized by dynamic light scattering (Zetasizer Nano-ZS90, Malvern, UK). High-resolution transmission electron microscopic (TEM) measurements of Pdots were recorded on a FEI Tecnai G2 Spirit T12 instrument (Waltham, MA, USA), operating at 120 kV. The UV-Vis absorption spectra were recorded using a DU-730 spectrophotometer (Beckman Coulter, Brea, CA, USA). The fluorescence spectra were obtained and calibrated using an FS5 fluorescence spectrophotometer (Edinburgh, Livingston, UK) using a 1 cm × 1 cm quartz cuvette. The fluorescence quantum yields were obtained using a Hamamatsu photonic multichannel analyzer C10027 equipped with a CCD and integrating sphere using pure DI water as the reference.

### 2.5. Cell Culture

Cells were provided by Procell Life Science and Technology Co., Ltd. (Procell; Wuhan, China). The cells were cultured in Dulbecco’s Modified Eagle Medium (DMEM) supplemented with 10% FBS, 1% penicillin, and streptomycin. The cells were incubated at 37 °C in a humidified environment with 5% CO_2_. The culture medium was changed every two days. Cells were detached with 0.25% trypsin-EDTA when the cells reached 80% confluence, then centrifuged at 800 rpm for 5 min. The pellets were resuspended in the culture medium and were subcultured in culture flasks.

### 2.6. Cell Viability

The cytotoxicity of Pdots was evaluated using a methyl thiazolyl tetrazolium (MTT) assay on mouse embryonic fibroblasts (NIH-3T3) cells. NIH-3T3 cells were cultured in 96-well plates at a density of 3000 cells per well. After a 24-h incubation, PFO/PFBT Pdots solutions at concentrations of 10, 20, 40, 50, and 60 µg mL^−1^ were prepared using two different methods and introduced into the medium (100 μL per well). For the control group, 100 μL of Pdot-free medium was used. Following another 24 h of incubation, each well was supplemented with 20 μL of MTT solution and incubated for an additional 4 h. The supernatant was then removed, and 200 μL of dimethyl sulfoxide (DMSO) was added to each well before being placed on a shaker for 10 min to fully dissolve the crystals. Absorbance was measured for each well using a microplate reader, taking readings of the optical density (OD) values at 490 nm. The cell survival rate in the control group was considered 100%.

### 2.7. Cell Imaging

Cells with a density of 8 × 10^4^ cells per well were seeded into a glass-bottomed culture dish and incubated overnight for adhesion under 5% CO_2_ at 37 °C. Then the cells were incubated with PFO/PFBT Pdots (20.0 µg mL^−1^) for 24 h. The cells were washed three times with PBS to remove extracellular Pdots. Cell images were acquired with a confocal laser scanning microscope (FV3000, Olympus, Shinjuku, Japan). The imaging objective was a UPLSAPO 40.0 oil-immersion objective.

### 2.8. Bioconjugation of Functionalized Pdots with Streptavidin

The bioconjugation reaction between carboxyl groups (-COOH) on Pdots and amine groups (-NH_2_) on streptavidin (SA) was catalyzed by 1-ethyl-3-(3-(dimethylamino)propyl) carbodiimide hydrochloride (EDC). Typically, 1 mL of Pdots (50 μg mL^−1^), 20 μL of HEPES buffer (1.0 M), 20 μL of 5% (wt/wt) PEG solution, 60 μL of SA solution (1.0 g L^−1^), and 20 μL of EDC solution (1.0 g L^−1^) were added to a 1.5 mL centrifuge tube, which was placed on a mixer at 20 r/min for 4 h. After the conjugation reaction, the product was mixed with PEG solution, HEPES solution, and Triton X-100 (Sigma-Aldrich, St. Louis, MO, USA) with a final concentration of 0.1% (wt/wt), 20 mM, and 0.2%, respectively, followed by purification using a 100 KD ultrafiltration centrifuge tube (ufc510096, Millipore, Darmstadt, Germany) to remove unconjugated free SA.

### 2.9. Subcellular Immunofluorescent Labeling and Imaging

BS-C-1 cells were plated at a density of 1 × 10^5^ cells per well in glass-bottom culture dishes and allowed to adhere overnight in a humidified incubator maintained at 37 °C with 5% CO_2_. Following adhesion, cells were subjected to cytoskeletal extraction using an extraction solution (0.1 M PIPES, 1 mM EDTA, 1 mM MgCl_2_, 0.2% Triton X-100, pH 7.2–7.4) for 30 s. Subsequent fixation was performed with 150 μL of a freshly prepared paraformaldehyde-glutaraldehyde solution (4% PFA, 0.1% GA in PBS) for 15 min at room temperature. After fixation, cells were permeabilized with 0.5% Triton X-100 in PBS for 5 min and blocked with 5% bovine serum albumin (BSA) in PBS for 30 min to prevent nonspecific binding. Microtubules were labeled by incubating with a biotinylated mouse monoclonal anti-α-tubulin antibody (Clone TU-01, Abcam ab74696, Cambridge, UK) diluted 1:100 in blocking buffer for 1 h at room temperature. High-resolution imaging was conducted using a confocal laser scanning microscope (Nikon AX, Melville, NY, USA) equipped with a UPLSAPO 100× oil-immersion objective (NA 1.4). All imaging parameters were kept constant between experimental conditions to ensure comparability of fluorescence signals.

## 3. Results and Discussion

### 3.1. Illustration of Semiconducting Pdots Prepared by Different Antisolvents

**Scheme 1:**
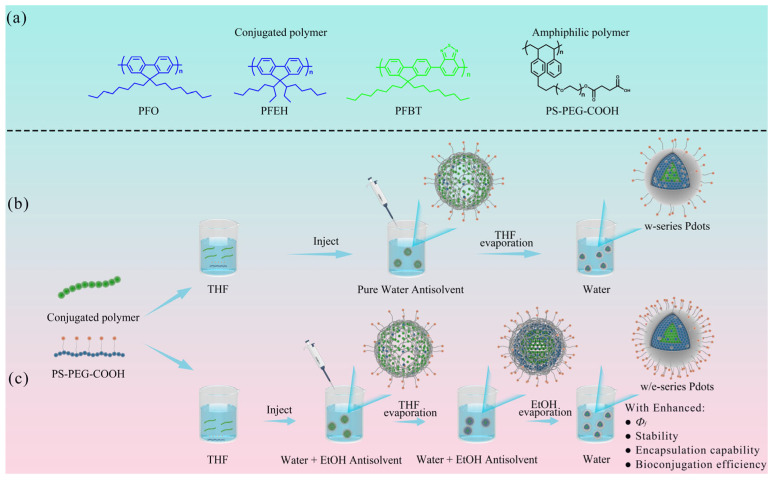
Chemical structures of PFO, PFEH, PFBT, and PS-PEG-COOH polymers (**a**) and schematic illustration of the w-series Pdots preparation processes using pure water antisolvent (**b**) and w/e-series Pdots preparation processes using water–ethanol mixed antisolvent (**c**).

As shown in Scheme 1a, we employ three classic CPs as research models in this study: PFO, PFEH, and PFBT. Here, PFO is a blue emissive polymer with three-dimensional (3D) organized crystal phase emission in Pdot or film state after solvent evaporation annealing treatment [36]. PFEH and PFBT are, respectively, blue and green emissive polymers, without any 3D ordered crystal phase emission in the Pdot or film state [37,38,39,40]. Classic PS-PEG-COOH was employed as the amphiphilic polymeric surfactant. Then, as shown in Scheme 1b,c, we prepared the corresponding Pdots by two different methods: (1) The traditional method uses pure water as the antisolvent, and the obtained Pdots are named as w-series Pdots; (2) The new method uses a water–ethanol mixture (1:1 by weight) as the antisolvent, and the produced Pdots are recorded as w/e-series Pdots.

### 3.2. Characterization of Semiconducting Pdots

As previously stated, we hypothesize that Pdots prepared using traditional nanoprecipitation methods do not exhibit an ideal core–shell nanostructure. As depicted in Scheme 1b, following the injection of THF solution, comprising conjugated polymers and PS-PEG-COOH, into the pure water antisolvent, these polymers quickly aggregate, forming larger, loosely structured NPs with an average hydrodynamic diameter of approximately 70 nm (Figure S1) by dynamic light scattering (DLS) measurement. This primarily occurs due to a drastic reduction in solubility. During the ensuing THF volatilization process, CP chains contract rapidly, and the particle size becomes smaller (Figure S2), forming a hydrophobic core intertwined with some of the PS-PEG-COOH chains. This likely happens because there is no ample time or space to expel most of the hydrophilic PEG chain segments from the core during the CP and PS hydrophobic chains’ reorganization process.

We posit that employing a water–ethanol mixture as an antisolvent might be an effective method to prepare Pdots with superior core–shell nanostructure (Scheme 1c). The rationale is as follows: (1) Ethanol is highly miscible with both THF and water solvents; (2) The boiling point of ethanol (78 °C) surpasses that of THF (66 °C) but remains lower than that of H_2_O (100 °C); (3) Due to the hydrogen bonding effect, ethanol possesses stronger intermolecular interactions with H_2_O than THF. Consequently, the volatilization sequence/speed at ~70 °C for their mixture is as follows: THF > ethanol > H_2_O. (4) As listed in Table S1, compared to water, ethanol exhibits superior solubility with the amphiphilic PS-PEG-COOH polymer, attributable to its enhanced swelling capacity for the hydrophobic PS component. Moreover, it exhibits very poor solubility with semiconducting polymers. Given these considerations, once the THF solution of CPs and PS-PEG-COOH is injected into the water–ethanol mixed antisolvent, THF is primarily the first organic component to be removed during the solvent volatilization process. Subsequently, the hydrophobic components (CP chains and the PS chain segment of PS-PEG-COOH), possessing low solubility in both ethanol and water, promptly coalesce to establish an inner core. The high solubility of PEG chain segments in both ethanol and water acts as a driving force, enabling the leaching of the PEG component from the hydrophobic core. Due to the presence of ethanol, capable of somewhat swelling the hydrophobic CP chains and PS chain segments, there is a more extended period and greater space available for the majority of the PEG chain segments to be excluded from the core during the CP and PS chain segment reorganization process. As the volatilization process progresses, the remaining organic solvent, primarily ethanol, is also gradually eliminated, causing the inner core to become increasingly compact. The alcohol/water-soluble PEG-COOH chain segments will subsequently accumulate on the surface of the hydrophobic core, forming the hydrophilic PEG-COOH shell. Owing to the amphiphilic nature of PS-PEG-COOH, the PS fragment coexists with the CPs in the inner core through hydrophobic interactions, while the PEG-COOH chain segment is extricated and coats the outer surface. Based on this progressive reprecipitation process, Pdots prepared by a water–ethanol mixed antisolvent may obtain a better core–shell structure. To validate our hypothesis, we selected three classic CPs (PFO/PFEH/PFBT) to prepare corresponding Pdots, utilizing either the conventional pure water antisolvent (w-series Pdots) or the novel water–ethanol mixed antisolvent (w/e-series Pdots). Subsequently, we characterized and examined the differences between these two series of Pdots.

**Figure 1:**
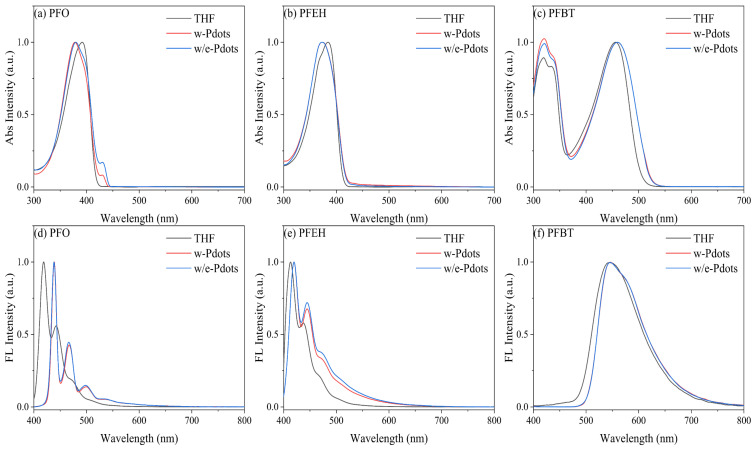
Normalized (**a**–**c**) absorption and (**d**–**f**) fluorescence spectra of PFO, PFEH, and PFBT in THF solution, and corresponding Pdots prepared by different ways at a concentration of 5.0 μg mL^−1^.

We commenced by investigating the photophysical properties of the three Pdots prepared through two distinct methods. Although the optical properties of Pdots primarily hinge on the molecular structure of the CPs [41], the stacked or arranged polymer chains in the aggregated state also influence Pdots’ photophysical properties to a certain degree. Figure 1 illustrates the normalized absorption and fluorescence spectra of PFO, PFEH, and PFBT Pdots prepared through two methods. For comparison, their absorption and emission spectra in a dilute THF solution are also presented.

As depicted in Figure 1a,b, the main absorption band of PFO/PFEH Pdots, attributable to the amorphous phase (glassy) polymer matrix, is broadened compared to their respective absorption band of random polymer chains in a THF solution, combined with blue-shifted absorption peak wavelength (*λ*_abs_) at around 365 nm. The hypsochromic shift of λ_abs_ could be attributed to the following possible reasons. Firstly, since the effective conjugation length (ECL) of polyfluorenes is estimated to be 12~64 fluorene units [42,43], the folding of PFO/PFEH polymer backbones within the small Pdots (R: ~25 nm) may shorten the ECL and result in blue-shifted absorption spectra. Secondly, the formation of H-aggregate (Figure S3), resulting in a hypochromic-shifted absorption band (Figure S4), occurs due to the notable planarity and high rigidity of the fluorene unit.

For the n-alkane substituted PFO Pdots, a prominent shoulder absorption peak at approximately 432 nm is evident, which emerges from the 3D ordered *β*-phase crystalline PFO, owing to the solvent annealing process [44,45,46]. The relative *β*-phase absorbance is further intensified for the w/e-PFO Pdots, primarily because there is sufficient time and available space to extract the PEG-COOH chain segment from the core, as previously discussed. A higher degree of chain segment rearrangement and a purer PFO content contribute to the formation of denser and more 3D ordered nanocrystals. For PFEH Pdots, the formation of 3D ordered nanocrystals is impeded due to the steric hindrance effect of the branched alkane chain. In addition to the face-to-face aligned *H*-aggregates, there may also be the formation of a few contents of head-to-tail aligned *J*-aggregate and/or low-dimensional ordered [47,48,49] microregions, resulting in slightly bathochromic-shifted tail absorption (Figure 1b).

For the PFBT Pdots, given that the benzothiadiazole (BT) unit serves as a potent electron-withdrawing group, the absorption band around 450 nm originates from the intramolecular charge transfer (ICT) transition from the weak electron-donating 9,9-dioctylfluorene group to the BT group. The presence of the BT unit results in the polymer having a shorter ECL, owing to its electron-deficient nature and the larger torsion angles with its adjacent units [50,51]. Therefore, the folding of the polymer chain in PFBT Pdots has a lesser impact on the ECL and does not lead to a blue shift of the absorption band. The large dihedral angle between BT and the 9,9-dioctylfluorene unit inhibits the PFBT polymer from forming a closely packed and 3D ordered crystalline phase under low-temperature annealing treatment (<120 °C) [49], which only permits the formation of low-dimensional oriented microregions with an extended conjugation length. And the 9,9-dioctylfluorene and BT units in adjacent chains tend to slip from each other to form a certain content of *J*-aggregates with head-to-tail alignment [38,50,52]. Both these aspects lead to an overall red-shifted absorption spectrum of PFBT Pdots relative to its absorption spectrum in diluted THF solution, as shown in Figure 1c.

As illustrated in Figure 1d, in contrast to PFO in THF solution, both w- and w/e-PFO Pdots display a red-shifted *β*-phase crystalline emission with a peak at around 438 nm. The *Φ*_f_ also significantly increases from 28.7% for w-PFO Pdots to 65.1% for w/e-PFO Pdots due to the suppressed self-quenching of fluorescence in the amorphous PFO host (Table 1). A key reason for the red-shifted emission and enhanced *Φ*_f_ is the effective Förster and through-bond energy transfer from the amorphous PFO matrix to the *β*-phase PFO self-dopant.

Neither the PFEH nor the PFBT Pdots exhibit crystalline phase emission. Compared to the fluorescence spectra in THF solution, the emission spectra of their Pdots exhibit a slight red shift of 7 nm and 2 nm, possibly due to extended conjugation length and/or the formation of *J*-aggregates. For the w/e-series of PFEH and PFBT Pdots, their *Φ*_f_ values are approximately 30% higher than those of the w-series Pdots. The *Φ*_f_ of PFEH Pdots increased from 33.2% to 41.6%, and the *Φ*_f_ of PFBT Pdots elevated from 25.3% to 33.9%. The higher *Φ*_f_ for w/e-series PFEH and PFBT Pdots is probably due to the following reasons: (1) A denser, higher-order amorphous core boosts the π-conjugation/rigidity of the polymer chains, thus mitigating non-radiative attenuation [53,54]; (2) A pure CP core eliminates the potential fluorescence quenching impact of the PEG chains themselves [55]. (3) The extraction of hydrophilic PEG chains minimizes the interface between the CP backbone and water molecules, subsequently reducing the surface free energy of the hydrophobic core-water interaction and suppressing the fluorescence quenching effect induced by the water medium [56,57].

Subsequently, we investigated the number-averaged size distribution by DLS measurements (Figure S2) and the surface Zeta potential (*ζ*) by electrophoretic mobility measurements of the two series of Pdots. As presented in Table 1, the two series of Pdots obtained exhibit similar diameters, approximately 25 nm. However, the *ζ* values for the w/e-series Pdots are generally lower compared to those of the w-series Pdots. This finding aligns with our hypothesis that Pdots prepared with a mixed antisolvent exhibit a preferable core–shell nanostructure. This nanostructure increases the carboxyl functional groups on the surface of Pdots, leading to a more negative charge on the outer surface of Pdot NPs. Theoretically, a higher absolute value of *ζ* increases the electrostatic repulsion between nanoparticles, thereby enhancing the colloidal stability of the Pdots. Additionally, a higher number of carboxyl functional groups on the Pdots surface improves the efficiency of bioconjugation with targets.

**Figure 2:**
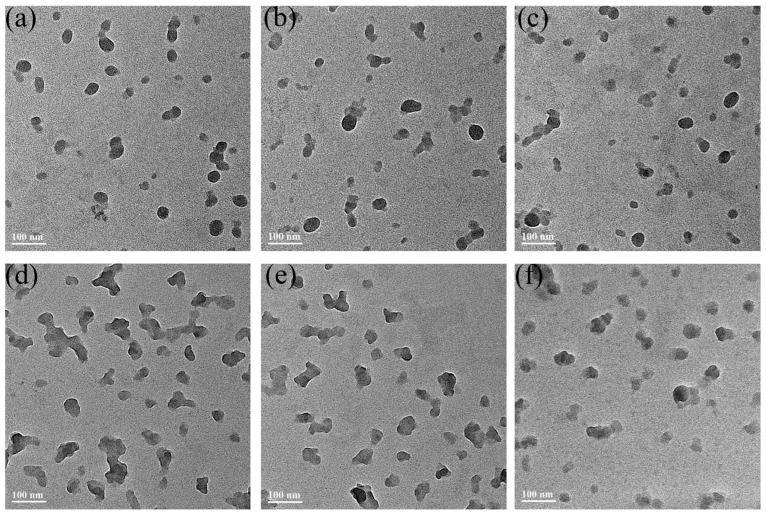
TEM images of Pdots prepared by two methods: (**a**–**c**) w-series Pdots, including (**a**) w-PFO, (**b**) w-PFEH, and (**c**) w-PFBT Pdots; (**d**–**f**) w/e-series Pdots, including (**d**) w/e-PFO, (**e**) w/e-PFEH, and (**f**) w/e-PFBT Pdots.

We also obtained surface profile images of various Pdots on a Cu-substrated carbon film using a high-resolution transmission electron microscope (TEM), as depicted in Figure 2. It can be seen that traditional w-series Pdots (Figure 2a–c) on the carbon film present a quasi-circular shape, while w/e-series Pdots (Figure 2e,f) illustrate a more irregular shape. We considered that PEG-COOH chains in the w-series of Pdots are more uniformly distributed throughout the nanoparticles. During the TEM sample preparation process, the rigid nanospheres tend to assume a quasi-circular shape after the evaporation of the water solvent. For each individual w-series Pdot NP, the distribution of its bright contrast shows no significant difference, which indicates the polymer density distribution is relatively homogeneous in the nanosphere.

For the w/e-series Pdots, anticipated to have a core–shell structure, most hydrophilic PEG-COOH chains reside at the interface between water and the hydrophobic nuclei, greatly diminishing the surface tension of the water dispersion medium, and thus enhancing the wettability of the aqueous dispersion on the carbon film surface. After the water volatilizes, the core–shell Pdots NPs can be better spread on the carbon film to form irregular morphology due to the collapse of the loose and soft PEG shells. Furthermore, there are distinct differences in brightness contrast in the TEM images of each w/e-series Pdots NP. The compact inner cores, having a higher polymer density, appear darker, whereas the loose PEG shells, with a lower polymer density, appear brighter.

**Figure 3:**
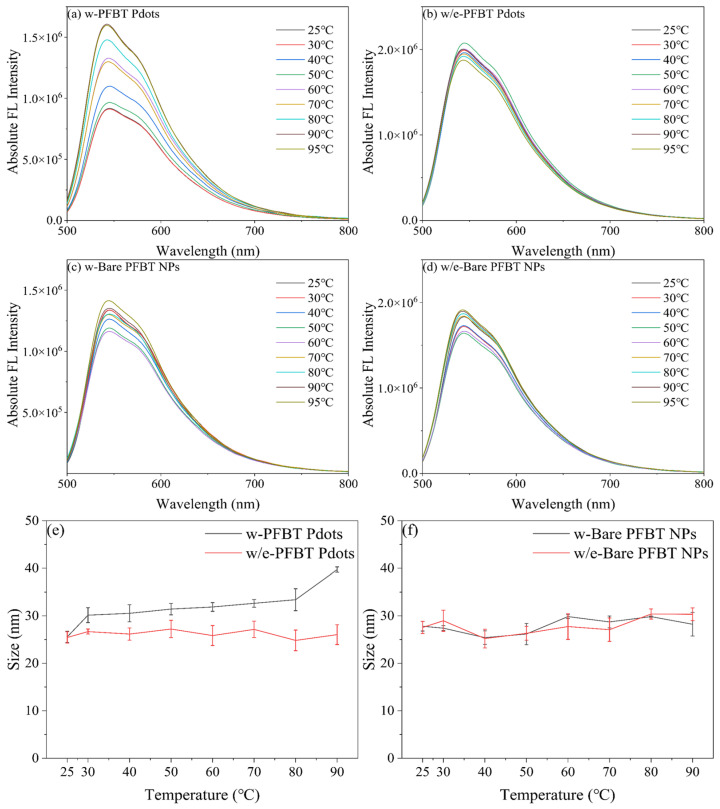
Temperature-dependent fluorescence spectra and diameters of PFBT-based nanoparticles. (**a**,**b**) Fluorescence spectra of (**a**) w-PFBT and (**b**) w/e-PFBT Pdots. (**c**,**d**) Fluorescence spectra of bare (**c**) w-PFBT and (**d**) w/e-PFBT NPs. (**e**,**f**) Temperature-dependent diameters of (**e**) w-PFBT and w/e-PFBT Pdots and (**f**) bare w-PFBT and w/e-PFBT NPs.

Next, we evaluated the temperature-dependent fluorescence spectra of Pdots NPs prepared by two different methods, using the PFBT polymer as a research model. As shown in Figure 3a,b, in contrast to the w/e-PFBT Pdots, the fluorescence intensities of the traditional w-PFBT Pdots fluctuate dramatically when the temperature rises from 25 °C to 95 °C. We thought that the large number of PEG chains embedded in the core causes the Pdots to swell with higher temperature, thereby influencing the aggregation-induced fluorescence quenching at different temperatures. To verify our conjecture, we prepared bare PFBT NPs without PS-PEG-COOH surfactant. As shown in Figure 3c,d, the bare PFBT NPs show quite weak fluorescence intensity variation with the temperature parameter. Additionally, both the w/e-series PFBT NPs (with and without PS-PEG-COOH) show a more stable fluorescence intensity compared to the w-series PFBT NPs.

We further studied the temperature-dependent diameters of the NPs prepared by two methods, including PFBT Pdots and bare PFBT NPs, using similar reprecipitation protocols. As shown in Figure 3e, the diameter of w-PFBT Pdots increased from ~25 nm to ~40 nm when the temperature rose from 25 °C to 90 °C. In comparison, the diameter of w/e-PFBT Pdots remains almost stable under different temperatures. On the other hand, bare PFBT NPs prepared by the two methods exhibit relatively stable particle sizes after heating up. The size of bare PS-PEG-COOH NPs dispersed in water remains quite stable at different temperatures as well (Figure S5), indicating the free PEG chains are well spread in aqueous solution.

All these phenomena guide that traditional Pdots, with embedded hydrophilic PEG chains in the hydrophobic core, easily undergo thermal expansion. Employing a water–ethanol mixed antisolvent can yield core–shell structured Pdots with stabilized fluorescence emission and particle size under high temperatures.

### 3.3. Encapsulation Capability of Semiconducting Pdots

**Figure 4:**
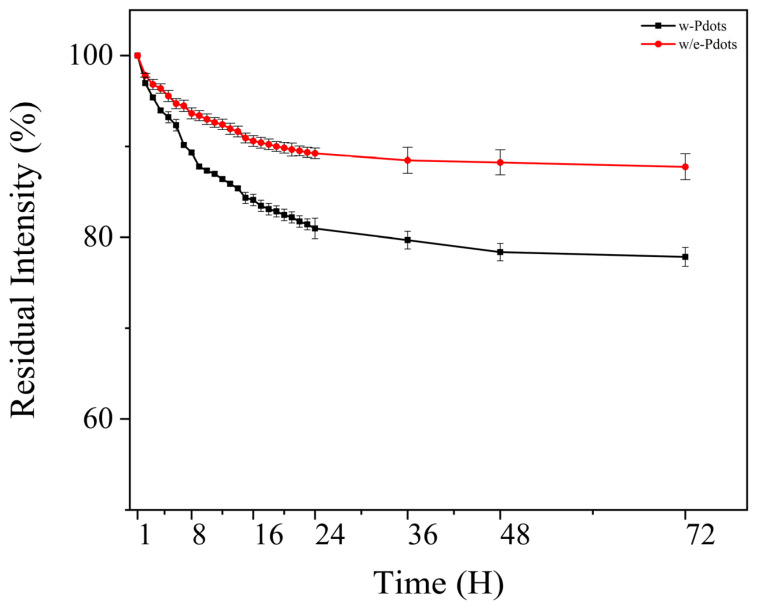
ICG dye residual percentage in w-PFH Pdots (black line) and w/e-PFH Pdots (red line) over 72 h (Normalized to initial absorbance = 100%).

To further evaluate the differences between w-Pdots and w/e-Pdots, we employed a dye leaching method to assess the encapsulation stability of the two types of PFH Pdots. The indocyanine green (ICG) dye covalently attached to a 1,2-Distearoyl-sn-glycero-3-phosphoethanolamine (DSPE) molecule was embedded into the Pdots by blending it (DSPE-ICG) with PFH polymer in the THF solvent during the nanoprecipitation process. Free DSPE-ICG was removed using ultrafiltration to ensure no free dye remained in the Pdot solution. The purified PFH Pdots were then dialyzed in ultrapure water using a dialysis bag (MWCO: 100,000) for 72 h. At specific time intervals, samples of the Pdot solution inside the dialysis bag were collected, and the residual ICG content was quantified by measuring its absorption intensity at 800 nm (Figure S6). Assuming the initial absorbance is 100%, the relative absorption intensity of ICG remaining in the two Pdots could be calculated as shown in Figure 4. After 72 h, the ICG leakage ratio from w/e-Pdots was only 12.2%, whereas that from w-Pdots reached 22.1%. This result demonstrates that the ICG encapsulated in w/e-Pdots exhibited a lower leakage rate compared to the one embedded in w-Pdots, further confirming that w/e-Pdots possess a more compact inner core. The different leakage kinetics also suggest that the w/e-Pdot effectively minimizes the molecular diffusion pathway, thereby improving the embedding stability of small cargoes. It is beneficial for those biomedical application scenarios that require long-term encapsulating capability of nanocarriers under physiological conditions, such as targeted therapy.

### 3.4. Förster Energy Transfer in a D-A Type Pdot System

**Scheme 2:**
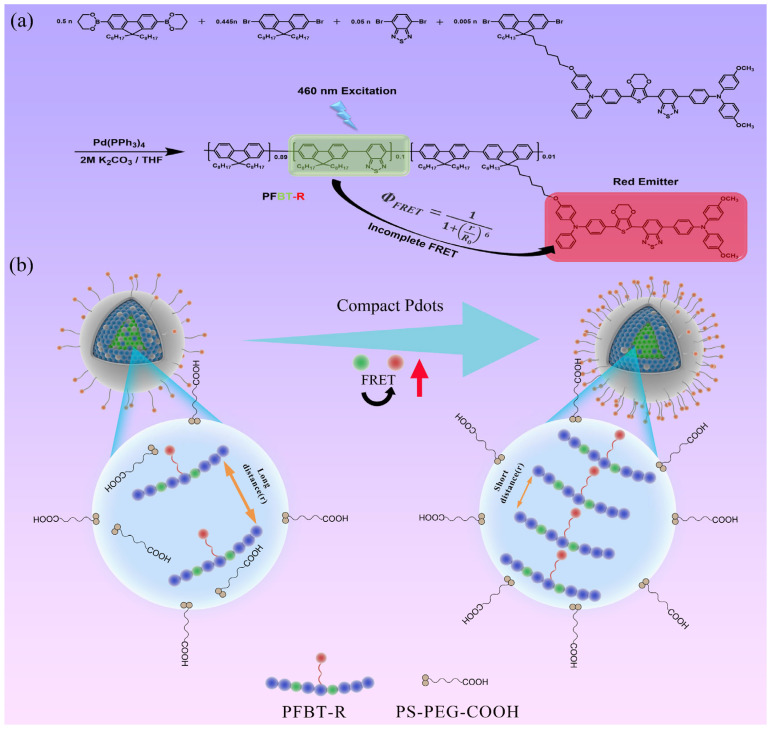
Schematic diagram of the synthesis of PFBT-R polymer and the FRET between green and red fluorophores (**a**) and the FRET difference in w- and w/e-PFBT-R Pdots (**b**).

To further verify that Pdots prepared using a water–ethanol mixed antisolvent possess a superior core–shell nanostructure with a compact hydrophobic inner core, we designed an energy donor-acceptor (D-A) PFBT-R Pdot system. The conjugated PFBT-R (Scheme 2a) polymer consists of a blue polyfluorene matrix (acting as dispersant), a green PFBT segment (acting as energy donor: D unit) in the backbone, and a red dye (acting as energy acceptor: A unit) attached in the side chain.

We chemically grafted the red emitter to prevent through-bond energy transfer from the green D to the red A fluorophore, ensuring only FRET between these two chromophores occurs when we directly excite the PFBT segment at a 460 nm wavelength (Scheme 2b). Meanwhile, the unwanted red dye leakage problem can be avoided thoroughly. The green PFBT content (feeding ratio) is up to 10 mol%, yielding a strong enough absorption cross-section from PFBT for direct excitation. The feeding ratio of the red emitter is only 1 mol% to control an incomplete energy transfer in this D-A type Pdot system. The red emitter has been reported in our previous work [35]. Here, we chose it as the A unit because its absorption spectrum (*λ*_abs_ = 530 nm) is well overlapped with the emission spectrum of the PFBT donor (*λ*_em_ = 535 nm), as shown in Figure S7, which benefits the efficient FRET between D-A units.

The Förster energy transfer efficiency (*Φ*_FRET_) between a pair of D-A fluorophores can be described by the following formula:

(1) $$\Phi_{\mathrm{FRET}}=\frac{1}{1+{\left(\frac{r}{R_{0}}\right)}^{6}}$$

Here, the *R*_0_ is the Förster radius, an average distance between D and A fluorophores for 50% *Φ_FRET_*. It depends on the molecular orientation of the fluorophores and the overlap integral between the donor emission spectrum and acceptor absorption spectrum. For a given D-A pair in our PFBT-R Pdot system, *R*_0_ could be roughly considered as a constant. Thus, the *Φ_FRET_* is inversely proportional to the sixth power of the average distance (*r*) between D and A units, making FRET extremely sensitive to small changes in distance. In this case, we can use *Φ_FRET_* (i.e., the relative fluorescence intensity of D-A units) to evaluate the *r* of D-A units in the PFBT-R Pdots prepared by different ways, thereby indirectly reflecting the stacking tightness of CP chains in hydrophobic nuclei.

**Figure 5:**
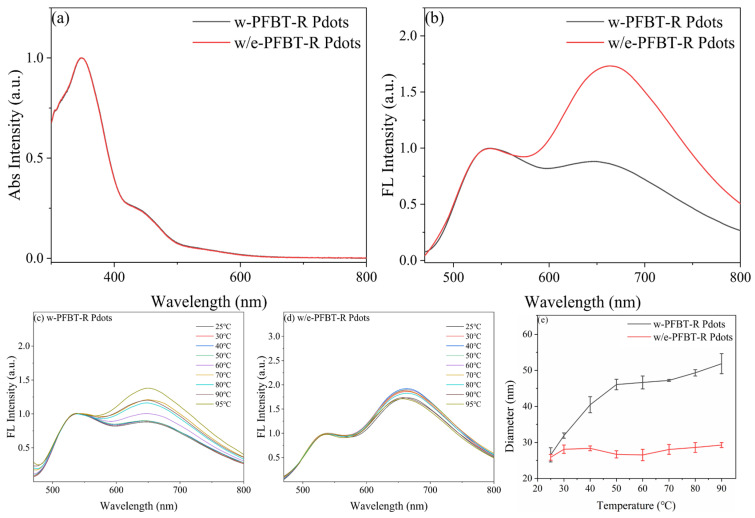
Characterization of PFBT-R Pdots prepared by two methods. (**a**) Normalized absorption and (**b**) fluorescence spectra of w-PFBT-R and w/e-PFBT-R Pdots at 25 °C. (**c**,**d**) Temperature-dependent fluorescence spectra of (**c**) w-PFBT-R and (**d**) w/e-PFBT-R Pdots. (**e**) Temperature-dependent diameters of w-PFBT-R and w/e-PFBT-R Pdots.

Two preparation methods were employed for PFBT-R Pdots, denoted as w-PFBT-R Pdots and w/e-PFBT-R Pdots, followed by the collection of their absorption and emission spectra. Figure 5a demonstrates identical absorption spectra for both types of Pdots, indicating no disparity in their components. Notably, as shown in Figure 5b, the relative intensity of the red emission band from w/e-PFBT-R Pdots is significantly amplified, over twice that of w-PFBT-R Pdots, indicating a more efficient FRET in w/e-PFBT-R Pdots. The *Φ*_f_ of the red emission (600–800 nm) is greatly improved from 18.2% to 32.6%. Therefore, one can deduce that the average distance (*r*) between the donor-acceptor (D-A) units is reduced in w/e-PFBT-R Pdots, which possess a purer and denser hydrophobic core, as depicted in Scheme 2b. As shown in Figure 5c–e, the w/e-PFBT-R Pdots exhibit more stable temperature-dependent fluorescence spectra and particle size, matching the performance of the aforementioned w/e-PFBT Pdots. These findings reinforce our initial anticipation that Pdots, prepared using a mixed antisolvent, exhibit an enhanced core–shell nanostructure.

### 3.5. Cytotoxicity and Living Cell Fluorescence Imaging

Cells subjected to 10% ethanol exposure succumbed within an hour, attributable to their low tolerance to ethanol [58]. Therefore, it is crucial to examine whether using a mixture of ethanol and deionized water as an antisolvent induces unwanted toxicity to cells. We first utilized an alcohol meter to assess the filtrate from the w/e-PFO and PFBT Pdots solution procured through centrifugal ultrafiltration, revealing negligible alcohol residue.

**Figure 6:**
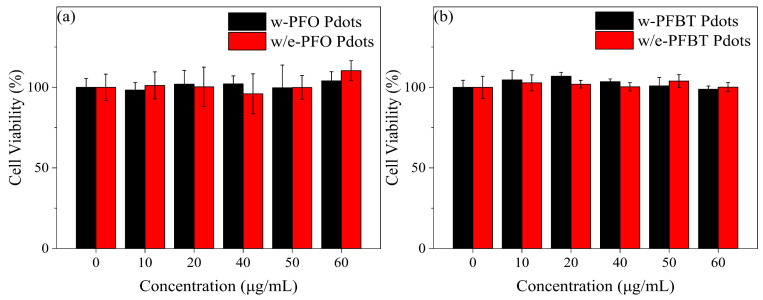
Cell viability of NIH-3T3 cells incubated with different concentrations of w-series and w/e-series Pdots: (**a**) w-PFO and w/e-PFO Pdots; (**b**) w-PFBT and w/e-PFBT Pdots.

Subsequently, we evaluated the cytotoxicity of the w- and w/e-series PFO/PFBT Pdots in NIH-3T3 cells using an MTT assay. Figure 6 shows that after 24-h incubation with Pdots prepared by the two methods, cell viability did not significantly decrease, and there was no marked difference in cell viability between the two methods. The experimental results demonstrate that upon ethanol removal, the Pdots prepared with a mixed antisolvent exhibited no additional toxicity to standard cell lines, and the novel preparation method preserved the high biocompatibility of Pdots without impeding subsequent biological applications.

**Figure 7:**
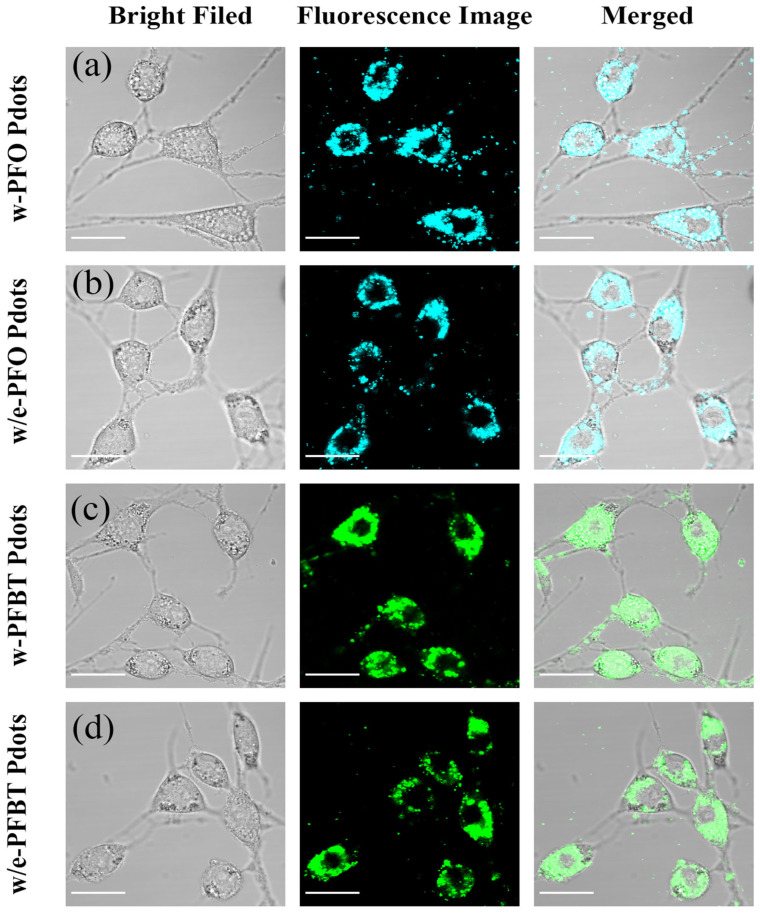
Bright-field, fluorescence, and merged CLSM images of NIH-3T3 cells incubated with w-series and w/e-series Pdots. (**a**,**b**) Cells incubated with (**a**) w-PFO and (**b**) w/e-PFO Pdots. (**c**,**d**) Cells incubated with (**c**) w-PFBT and (**d**) w/e-PFBT Pdots (Scale bar: 20 μm).

To explore whether the new preparation method affects the endocytosis of Pdots by cells, we incubated NIH-3T3 cells with w- and w/e-series PFO/PFBT Pdots for 24 h and then performed fluorescence imaging. Under confocal laser scanning microscopy, both series of PFO and PFBT Pdots were visibly internalized by the cells, displaying bright fluorescence in the cytoplasm. There was no significant difference in fluorescence intensity, with PFO Pdots manifesting as blue light, and PFBT Pdots as green light (Figure 7).

Furthermore, bright-field images illustrated that cells incubated with the four types of Pdots maintained good health, and the Pdots prepared with the mixed antisolvent did not trigger evident signs of apoptosis or inflammation. Based on the outcomes of MTT and cell imaging, it is evident that the process of nanoprecipitation utilizing the mixed antisolvent does not influence the effect of Pdots on cells. The Pdots maintain good biocompatibility, catering to the requirements of subsequent research and enabling conditions for in vivo applications.

### 3.6. Intracellular Cytoskeleton Immunofluorescent Labeling and Imaging

**Figure 8:**
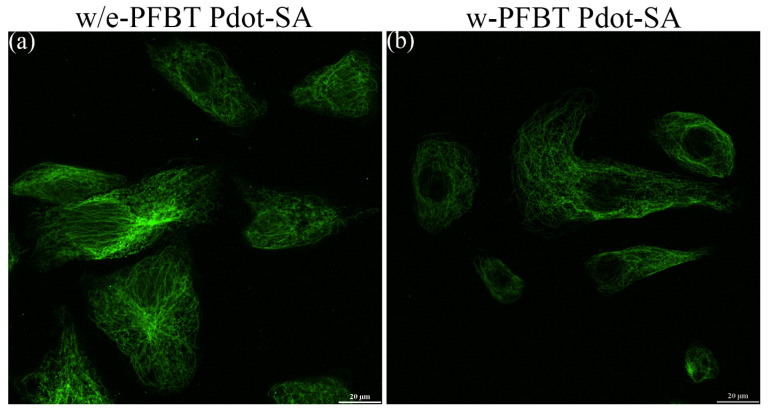
Intracellular cytoskeleton immunofluorescent labeling and imaging of BS-C-1 cells using (**a**) w/e-PFBT Pdot-SA and (**b**) w-PFBT Pdot-SA. (Scale bar: 20 μm).

In addition to fluorescence labeling cell profiles through endocytosis, using the Pdots-SA-biotin-antibody–antigen system, specific immunofluorescence labeling of subcellular organelles can also be achieved [59,60]. Therefore, we chemically incorporated SA with w-Pdots and w/e-Pdots through EDC-catalyzed reaction between carboxyl group (-COOH) groups on Pdots and amine (-NH_2_) groups on SA molecules to perform subcellular microtubule labeling in BSC-1 cells. In this way, we can compare and study the bioconjugation efficiency of two types of Pdots with the target SA molecule. After bioconjugation with SA, both w-PFBT Pdots and w/e-PFBT Pdots exhibited a moderate increase in hydrodynamic diameter value and a reduction in Zeta potential (*ζ*) value, as determined by DLS and electrophoretic mobility measurements (Table 2). Notably, w/e-PFBT Pdots demonstrated a more significant increase in average diameter (9.1 nm vs. 6.4 nm) and decrease in surface charge (8.1 mV vs. 5.7 mV) compared to that of w-PFBT Pdots, consistent with their higher surface carboxyl group (-COOH) density. These phenomena indicate that w/e-PFBT Pdots bind more efficiently with the SA target. These results validate the superior functionalization capacity of w/e-Pdots, attributable to their well-engineered surface architecture, which provides greater accessibility for covalent coupling while maintaining nanoparticle stability. For intracellular cytoskeleton immunofluorescent labeling and imaging, BS-C-1 cells were sequentially incubated with biotinylated mouse anti-α-tubulin antibody and the PFBT Pdot-SA conjugates. Then, they acquired the fluorescence images using confocal laser scanning microscopy (Figure 8). It can be seen from Figure 8a that the individual tubular structures labeled by w/e-PFBT Pdot-SA complex were more clearly resolved than the tubular structures labeled with w-PFBT Pdot-SA complex (Figure 8b). This result further supports our speculation that the w/e-PFBT Pdots prepared using water–ethanol mixed antisolvent possess a better core–shell nanostructure with a higher density of surface-exposed carboxyl groups, which is beneficial for improving the chemical bioconjugation efficiency with target molecules, such as SA in this study.

## 4. Conclusions

In this study, we optimized the preparation method of Pdots by simply replacing the deionized water antisolvent in the nanoprecipitation process with a mixed antisolvent of deionized water and ethanol. This strategy can be conveniently and widely adopted for the preparation of Pdots. Compared to the traditional method, this advanced nanoprecipitation method endows the Pdots with an optimized core–shell nanostructure and superior fluorescence quantum efficiency. It might be a universal approach to obtain more efficient Pdot fluorescent probes, especially for dopant/host-based Pdot systems, due to boosted *Φ*_FRET_, whether the dopant is artificially introduced or in situ self-generated (aggregation-induced emissive species with a lower energy gap). The Pdots prepared by the novel method exhibit both luminescent spectral stability and dimensional stability, regardless of ambient temperature. Furthermore, a compact inner core reduces the risk of leakage and is better suited for encapsulating small organic luminescent dyes or highly hydrophobic drugs with small molecular volumes. An optimal PEG shell provides a stronger coating of the CP core, with more outer-surface functional groups, which is beneficial for more efficient bioconjugation with target molecules. Generally, this research is anticipated to foster the advancement of Pdots production technology and provide additional insights into the true microstructure of Pdots and how it connects to their macroscopic properties.

## Figures and Tables

**Table 1:** Photophysical properties, diameter (*R*), and Zeta potential (*ζ*) of different Pdots. [a] Measured in 0.005 g L^−1^ Pdots solution. [b] Absolute fluorescent quantum yield. [c] Measured by DLS. Averaged values for three independent batches.

Pdots	Antisolvent	*λ*_abs_ [a] (nm)	*λ*_em_ [a] (nm)	*Φ*_f_ [b] (%)	*R* [c] (nm)	*ζ* (mV)
PFO	Water	378	438	28.7 ± 3.4	26.0 ± 1.9	−22.1 ± 0.8
	Water/EtOH	380	439	65.1 ± 2.2	25.3 ± 2.0	−23.3 ± 1.0
PFEH	Water	374	420	34.2 ± 1.8	28.1 ± 0.6	−21.0 ± 0.5
	Water/EtOH	374	420	41.6 ± 3.0	25.8 ± 0.8	−22.9 ± 1.9
PFBT	Water	457	545	25.3 ± 3.0	23.5 ± 0.9	−21.1 ± 0.5
	Water/EtOH	457	545	33.9 ± 5.6	23.3 ± 0.8	−22.3 ± 4.6

**Table 2:** Diameter and Zeta potential of PFBT Pdots and PFBT-SA. [a] Differences between PFBT Pdots-SA and PFBT Pdots. Averaged values for three independent batches.

	w-PFBT	w-PFBT-SA	Δ Value [a]	w/e-PFBT	w/e-PFBT-SA	Δ Value [a]
*R* (nm)	26.5 ± 1.5	32.9 ± 2.2	6.4	25.3 ± 0.9	34.4 ± 2.0	9.1
*ζ* (mV)	−21.1 ± 0.5	−15.4 ± 1.5	5.7	−23.3 ± 0.8	−15.2 ± 0.6	8.1

## Data Availability

Data will be made available on request.

## Supplementary Materials

The following supporting information can be downloaded at https://www.mdpi.com/article/10.3390/bios16060308/s1. Figure S1: Number-averaged size distribution investigated by DLS of PFO, PFEH and PFBT nanoparticles after THF solution initially injected into different antisolvents; Figure S2: Number-averaged size distribution investigated by DLS of PFO, PFEH and PFBT Pdots prepared by two methods; Figure S3: Illustration of the formation of *β*-phase crystalline PFO and *J*/*H*-Aggregate of PFO/PFEH polymer in nano-aggregation state; Figure S4: Illustration of the formation mechanism of non-emissive *H*-aggregate with blue-shifted absorption band and emissive *J*-aggregate with red-shifted absorption band; Figure S5: Temperature-dependent diameters of bare PS-PEG-COOH NPs prepared by two methods; Figure S6: Normalized absorption spectra of DSPE-ICG loaded w-PFH Pdots and w/e-PFH Pdots solution in dialysis bag at specific time intervals, and the enlarged absorption region of ICG dye; Figure S7: The normalized emission spectrum of PFBT10 polymer and the absorption spectrum of the red emitter in diluted THF solution; Table S1: Solubility of PFO/PFEH/PFBT/PS-PEG-COOH in Water/Ethanol/THF.
