# Supplementary material for: Creating Semiconducting Polymer Dots with Enhanced Performance Through a Simple Mixed Antisolvent Approach
Source: Biosensors (Basel). 2026 May 27;16(6):308. doi: 10.3390/bios16060308 (PMC13296412; doi:10.3390/bios16060308)
Supplement: Supplementary file 1 [file biosensors-16-00308-s001.zip › biosensors-4279798-supplementary.pdf]

Supplementary Materials

Creating Semiconducting Polymer Dots with Enhanced Performance Through a Simple Mixed Antisolvent Approach

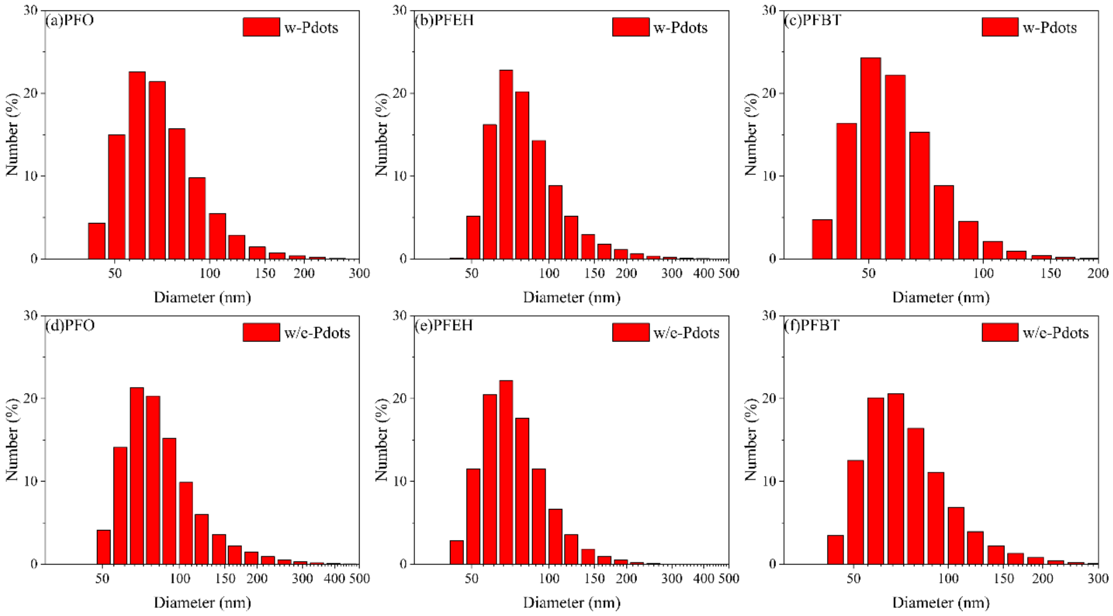

Figure S1. Number-averaged size distribution investigated by DLS of (a, d) PFO, (b, e) PFEH and (c, f) PFBT nanoparticles after THF solution initially injected into different antisolvents.

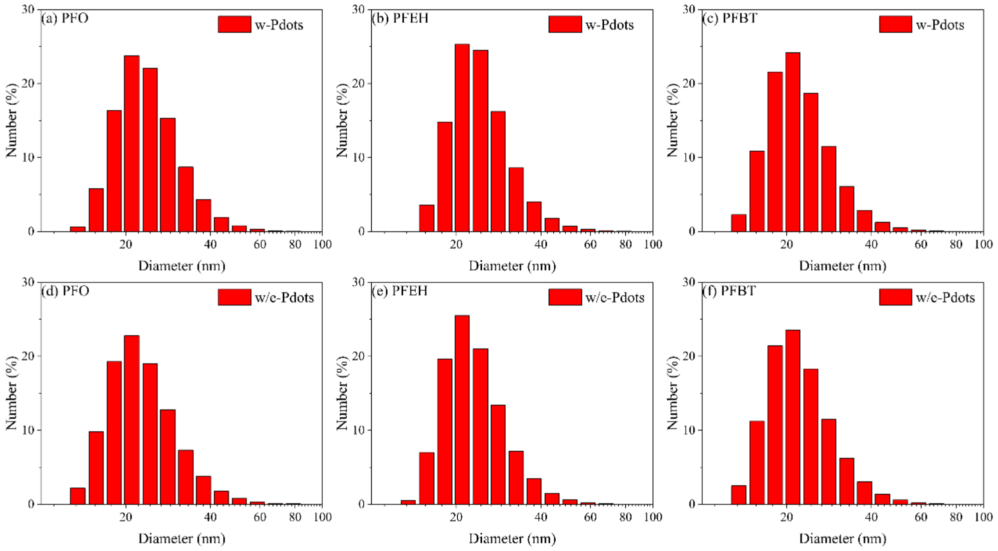

Figure S2. Number-averaged size distribution investigated by DLS of (a, d) PFO, (b, e) PFEH and (c, f) PFBT Pdots prepared by two methods.

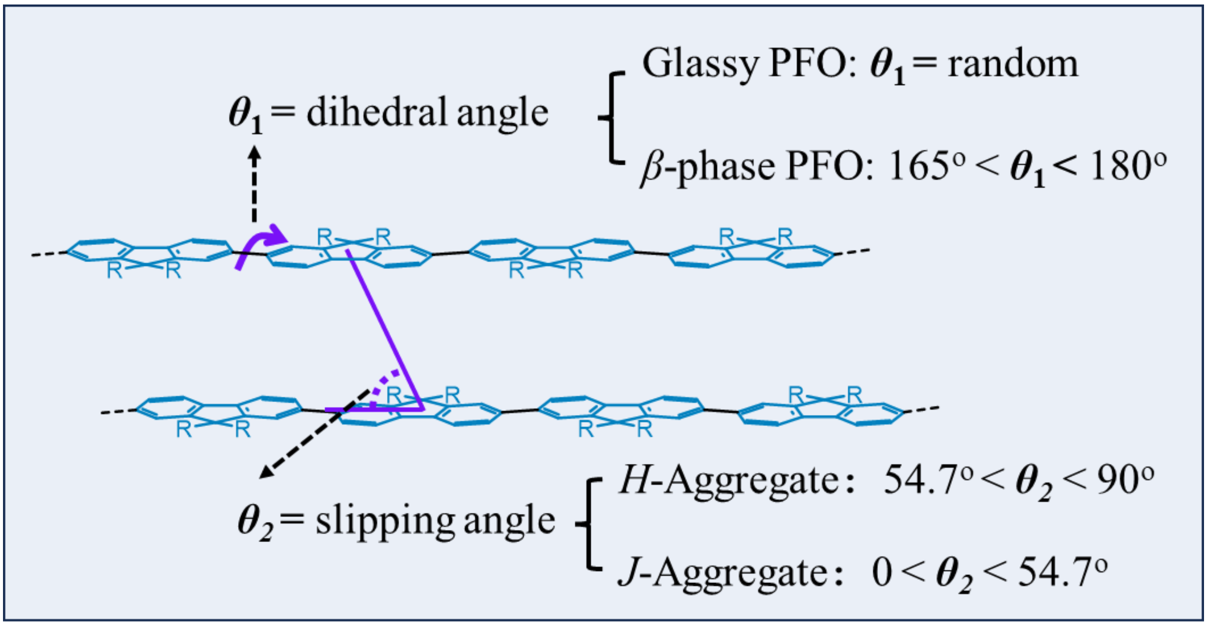

Figure S3. Illustration of the formation of *β*-phase crystalline PFO and *J*/*H*-Aggregate of PFO/PFEH polymer in nano-aggregation state.

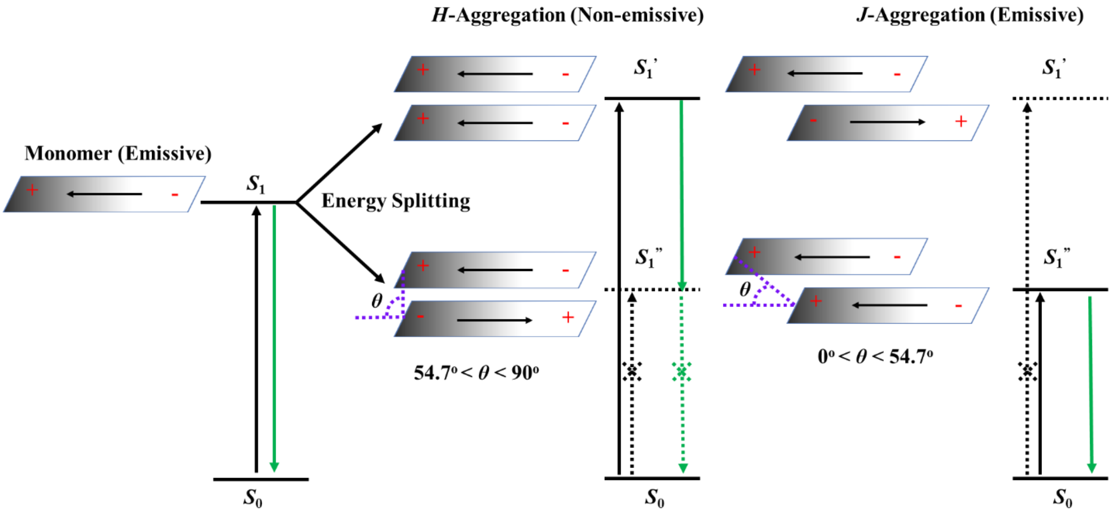

Figure S4. Illustration of the formation mechanism of non-emissive *H*-aggregate with blue-shifted absorption band and emissive *J*-aggregate with red-shifted absorption band.

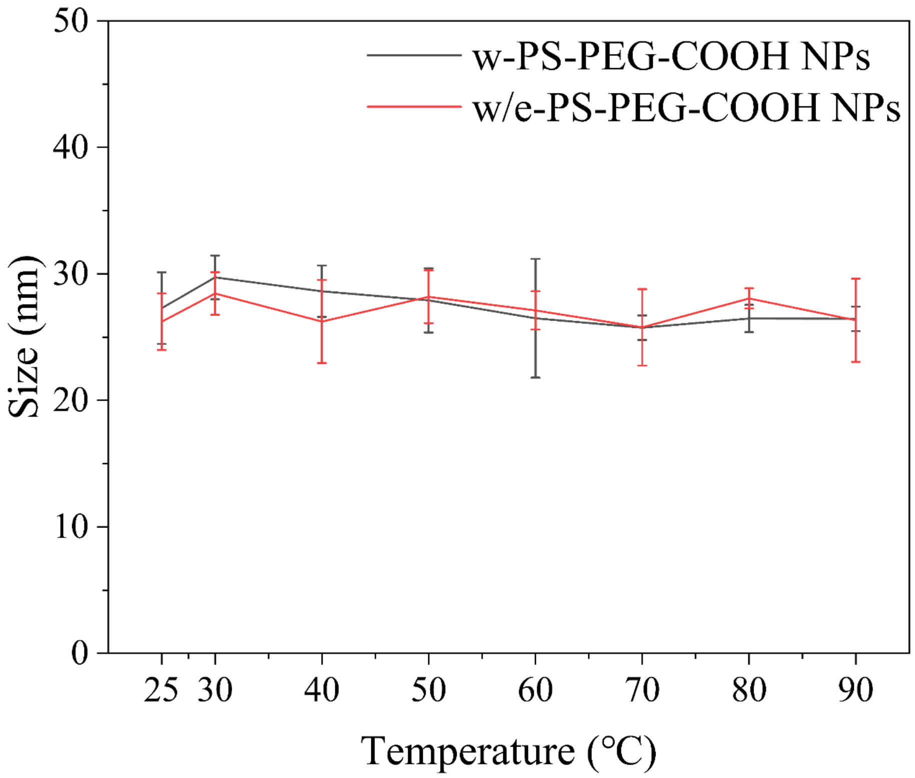

Figure S5. Temperature-dependent diameters of bare PS-PEG-COOH NPs prepared by two methods.

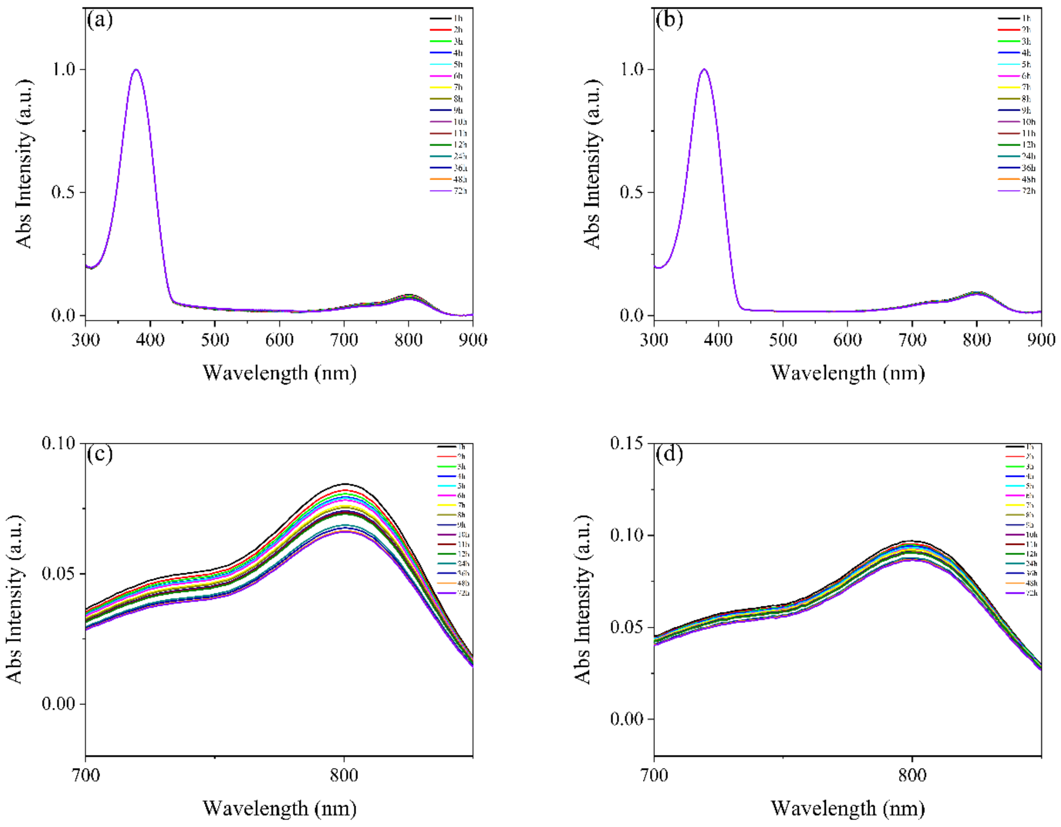

Figure S6. Normalized absorption spectra of DSPE-ICG loaded w-PFH Pdots(a) and w/e-PFH Pdots (b) solution in dialysis bag at specific time intervals; (c) and (d) are the enlarged absorption region of ICG dye of (a) and (b), respectively.

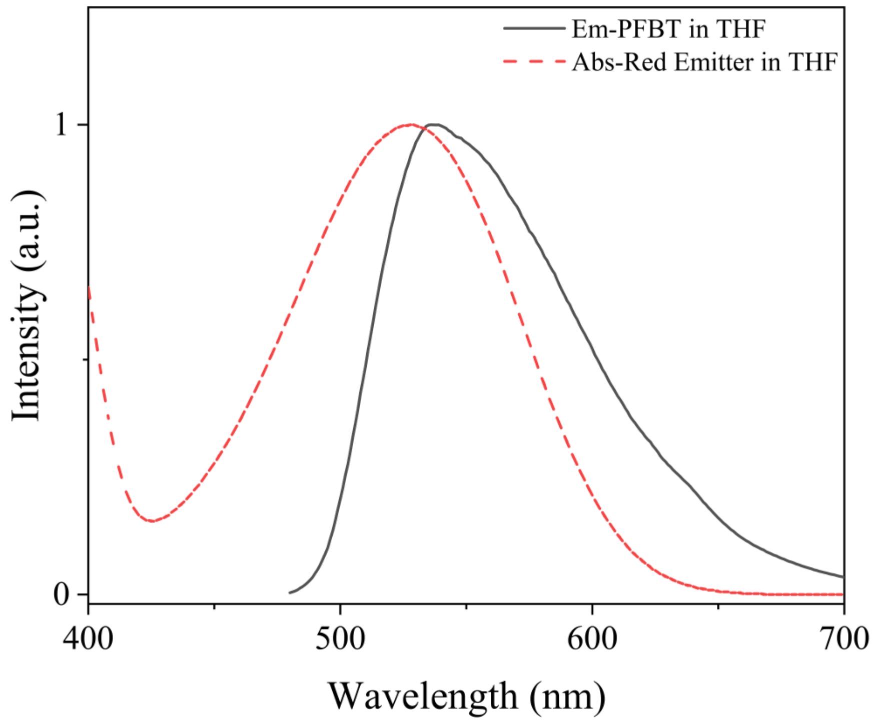

Figure S7. The normalized emission spectrum of PFBT10 polymer and the absorption spectrum of the red emitter in diluted THF solution (0.005 mg mL^{-1}).

Table S1. Solubility of PFO/PFEH/PFBT/PS-PEG-COOH in Water/Ethanol/THF.

	Di-water	Ethanol	THF
PFO	-	< 2.5 ug mL^{-1}	~ 38 mg mL^{-1}
PFEH	-	< 2.5 ug mL^{-1}	~ 110 mg mL^{-1}
PFBT	-	< 2.5 ug mL^{-1}	~ 22 mg mL^{-1}
PS-PEG-COOH	< 0.05 mg mL^{-1}	~ 1.5 mg mL^{-1}	~ 175 mg mL^{-1}
